# Validation of the Psychometric Properties of the Practice Environment Scale of Nursing Work Index in Primary Health Care in Portugal

**DOI:** 10.3390/ijerph18126422

**Published:** 2021-06-14

**Authors:** Pedro Lucas, Elvio Jesus, Sofia Almeida, Beatriz Araújo

**Affiliations:** 1 Center for Interdisciplinary Research in Health, Instituto Ciências da Saúde, Universidade Católica Portuguesa, Rua de Diogo Botelho, 1327, 4169-005 Porto, Portugal; ejesus@porto.ucp.pt (E.J.); spalmeida@porto.ucp.pt (S.A.); baraujo@porto.ucp.pt (B.A.); 2 Nursing Research, Innovation and Development Center of Lisbon, Nursing School of Lisbon, 1600-190 Lisbon, Portugal

**Keywords:** community health nursing, health facility environment, nursing, primary health care, psychometric properties, quality of care, validation study, work environment

## Abstract

Studies related to the work environment in primary health care are scarce in the literature. The present study aimed to validate the psychometric properties of the Practice Environment Scale of Nursing Work Index (PES-NWI) in primary health care (PHC) and to evaluate its construct validity through exploratory and confirmatory factor analyses in a sample of Portuguese nurses. A quantitative, cross-sectional, and validation study design was implemented. Methods: The sample consisted of 1059 nurses from the PHC units of all 55 health center groups (HCGs) in mainland Portugal, 15 health centers in the Autonomous Region of Madeira, and 6 health centers in the Autonomous Region of the Azores. The study tested different structural models using exploratory and confirmatory factor analysis techniques. The reliability of the scale was tested by determining Cronbach’s alpha coefficient. Results: The internal consistency of the PES-NWI was 0.91. Exploratory and confirmatory factor analyses were performed on the PES-NWI model in PHC with five factors: NPOA, NFQC, NMALSN, SRA, and CNPR. The results show that the scale presents acceptable fit quality indexes in the final factorial solution and adequate convergent validity. Conclusion: The PES-NWI in PHC has an adequate, robust, and reliable five-factor structure. The scale is valid and can be used in clinical practice, nursing management, and PHC research.

## 1. Introduction

Knowledge and scientific evidence about nursing practice environments (NPEs) in the context of primary health care (PHC) are scarce [1,2,3,4] compared with those in other contexts, such as hospitals. According to Lake [5], NPEs are defined as the organizational characteristics of a work context that facilitate or constrain the professional practice of nurses. NPEs are vital for the success of health systems [6]. They relate to the quality of nursing care, job satisfaction, patient safety, the effectiveness of client care, and the efficiency of organizations [1,5,7,8].

Promoting the quality of care offered by nurses, and thus contributing to the improvement of clinical practice contexts, is a vital factor of NPEs. The quality of nursing care is an essential element in the profession; it refers to, among other aspects, the direct relationship between the client and the nurse. It depends on several factors, particularly the NPEs themselves [1,9]. In primary health care, NPEs are a key factor in ensuring quality nursing care with positive results for clients, teams, and organizations.

A favorable NPE leads to better results for clients, as it is an essential factor to increase nurse satisfaction [5,10], and it is fundamental to ensure that teams have an appropriate number of nurses and other staff members [1,8]. A favorable NPE is characterized by adequate human resources and materials, active participation of nurses in the governance, quality of care and nursing care, and good relationships among distinct groups of health service practitioners [5,6]. According to the scientific evidence produced in the last few decades, favorable NPEs have a significant impact on the levels of quality and safety of customer care; health professionals’ well-being, quality, and productivity; and the effectiveness of health services, organizations, and systems [6].

On the contrary, a poor NPE, with little support from management, weak leadership, and poor multidisciplinary relationships, is associated with a decrease in the quality of nursing care, adverse effects on clients [4] (such as malpractices, increased mortality and complications, readmissions for complications, increased health care costs, and ineffective care), conflicts and stress among health care professionals [9], job dissatisfaction, and increased turnover of nurses [1]. All of these aspects strongly contribute to patient dissatisfaction with the care that they receive.

A safe NPE is characterized by reliable professional relationships among its members, management support for practitioners, and a balanced work schedule [1,11,12]. It is also characterized by an appropriate balance between nurses’ workload and skills, adequate time to answer a client’s needs, professional autonomy, sufficient resources, and opportunities for professional progression [1,11,12].

Nurse managers perform a vital role in the creation of a favorable and positive NPE [11] and the promotion of quality care [1,7]. They can also provide the necessary tools for nurses’ professional development and future managers [13]. Nursing leadership performs a significant role in the quality care provided to the client, which involves four fundamental activities: facilitation of effective continuous communication, strengthening of interprofessional and interprofessional relationships, construction and preservation of teams, and involvement of peers [7]. Leaders influence NPEs [1,14] and the quality of nursing care [7]. Nurses, as leaders, are crucial for improving communication among teams to reach goals and to increase the quality of care, patient safety, and health innovation [7,15].

Without skills and proper knowledge, it becomes difficult for nursing leaders to maintain a favorable practice environment [1,11]. A nurse manager is a driver of change toward excellence by organizing existing resources and creating a safe environment for nursing care [1].

Thus, there is a need to study the NPE at other levels of the health care system, in addition to the hospital context [1], particularly in primary health care, mainly because there are few studies in this context [1].

For NPE evaluation, the measuring instrument most used in scientific research is Lake’s [5] PES-NWI [13,16,17,18]. This scale allows investigators to identify the factors that contribute to the NPE for optimal results for nurses and clients and produces solid and comparable data [5,16]. Its use is recommended by several world organizations associated with quality evaluation, such as the National Quality Forum, The Joint Commission on Accreditation of Healthcare Organizations, and the National Database of Nursing Quality Indicators [16,19].

In Portugal, the PES-NWI was translated and validated by Amaral et al. [19] and used in several studies in the hospital context [10,20,21,22,23], but no such research has been performed in PHC.

According to a recent scoping review by Lucas and Nunes [1] about the NPE in PHC, the PES-NWI was the most commonly used NPE measuring instrument among the reviewed studies [3,13,24,25,26,27,28,29]. According to this literature review, the PES-NWI is an internationally validated instrument that measures the fundamental variables of the NPE in PHC and allows for evaluating, analyzing, and redirecting nurses’ practices in this context of care [1,25].

Other instruments measure the NPE, but the PES-NWI is the most frequently used because it enables the evaluation of different objectives owing to its diversity of items, its good psychometric properties, and the comparability of results between studies and countries [5,18,30]. The PES-NWI is one of the most widely used instruments to measure the quality of NPEs [16] and relates aspects of nursing stability to patient safety and the quality of nursing care [10]. This scale evaluates several dimensions of the NPE through nurses’ opinions about the conditions in which they work, thereby providing a better understanding of possible challenges for human resource management and health governance [10].

The PES-NWI has been translated, validated, and used in different cultural/language contexts: Portuguese [19], Brazilian Portuguese [31], Spanish [32], French [33], Belgian [34], Danish [35], Polish [36], Icelandic [37], Cypriot [38], Chinese [39], Korean [40], Australian [41], Colombian [42], and Japanese [43]. All of these studies were carried out in hospitals. The only PES-NWI validation study in primary health care that is known is De Pedro-Gómez et al. [25]. Thus, the present study is of great importance, as it is the second PES-NWI study in the world carried out in primary health care.

This study aimed to evaluate the psychometric proprieties of the PES-NWI in PHC through exploratory and confirmatory factor analyses in a sample of Portuguese nurses.

## 2. Materials and Methods

### 2.1. Study Design

A quantitative, cross-sectional, and validation study was designed to evaluate the psychometric properties of the Portuguese version of the PES-NWI [19] in primary health care.

### 2.2. Method

The initial protocol of the RN4CAST Portugal study was replicated in 2018. The study design was quantitative and cross-sectional and aimed to validate the psychometric properties of the PES-NWI in primary health care in mainland Portugal and in the autonomous regions of Madeira and Azores.

#### 2.2.1. Data Collection and Procedure

Data were obtained online from November 2017 to May 2018. The study was facilitated by a Portuguese university by the coordination team of the RN4CAST study and the Portuguese Nurses Order through their institutional websites. The invitation was e-mailed to nurses affiliated with the Portuguese Nurses Order with a link to complete the instrument developed by the RN4CAST consortium, called the RN4CAST Nurse Survey Instrument. It was composed of sociodemographic variables (age, gender, professional nursing experience, and academic education), the PES-NWI, burnout scales, quality of nursing care, job satisfaction, turnover intention, patient safety, workload, and endowments. This article focuses on the psychometric evaluation of the PES-NWI in primary health care owing to a lack of studies in this context and its international importance.

The PES-NWI comprises 31 items evaluated on a Likert scale with four response options from 1 to 4 (1 = completely disagree; 2 = disagree; 3 = agree; 4 = completely agree). The Portuguese version of the PES-NWI covers the following dimensions: (1) “Nurse Participation in Hospital Affairs” (9 items); (2) “Nursing Foundations for Quality of Care” (10 items); (3) “Nurse Manager Ability, Leadership, and Support of Nurses” (5 items); (4) “Staffing and Resource Adequacy” (4 items); and (5) “Collegial Nurse–Physician Relations” (3 items) [19].

#### 2.2.2. Participants

The inclusion criteria of the study were nurses who work in health centers in Portugal and who agreed to complete the questionnaire.

In this study, 1059 nurses working in primary health care in Portugal answered the questionnaire. These nurses worked in 55 health center groups from the Portuguese mainland, 15 health centers in the Autonomous Region of Madeira, and 6 health centers in the Autonomous Region of the Azores. The respondents in Portugal answered the second edition of the online questionnaire developed by the RN4CAST@pt study in 2018.

### 2.3. Analysis

A descriptive and comparative analysis of the variables was carried out. The continuous variables were described by the mean and standard deviation, while the categorical variables were described by relative and absolute frequencies.

The exploratory factor analysis (EFA) technique was applied to generate possible factorial structures. The sample matched all items on the scale, so Little’s MCAR test was not performed.

To perform the EFA, we extracted the main components using the Varimax rotation method. For the analysis of the adequacy of data for EFA, we used two tests: The Kaiser–Meyer–Olkin (KMO) test, the value of which must be higher than 0.5, and Bartlett’s sphericity test. Both indicate the suitability of data for factorial analysis [44]. For the factor analysis, we retained the items with a factor loading above 0.4 [44].

We used the Kaiser criterion to extract the factors using the Varimax rotation technique. The total variance explained by the results was analyzed.

We determined the internal consistency and the reliability of the instrument using Cronbach’s alpha coefficient (α) and the composite reliability. These values may vary from 0 to 1, with 0.70 being the minimum value for acceptable reliability [44].

To evaluate the quality of the model adjustment obtained through EFA, a confirmatory factor analysis (CFA) was carried out using AMOS software (version 26.0), IBM Company (IBM Corp, Armonk, NY, USA). CFA was performed using the maximum likelihood method, which assumes the independence of observations, multivariate normality, and absence of outliers. The normal distribution of the variables was analyzed by the asymmetry coefficient (Sk) and kurtosis (Ku). Outliers were evaluated using the squared Mahalanobis distance (D^2^) [45]. 

To evaluate the global adequacy of the model in CFA, the following indexes were used: the goodness-of-fit index (GFI) and the comparative fit index (CFI), in which a score higher than 0.90 reveals a good fit [45]; the root mean square error of approximation (RMSEA), with an acceptable score between 0.05 and 0.08; and the standardized root mean square residual (SRMR), with an acceptable score below 0.08 [45]. The model with the lowest (L) expected cross-validation index (L)ECVI represents the best fit. For this purpose, we used the modification indexes provided in AMOS, as well as in theoretical considerations [45]. Mardia’s coefficient was not used because this significance test alone is not a practical assessment of normality, especially in a structural equation model [46].

The structural validity of the scale was first tested using the EFA and then using structural equation models. CFA was tested using factorial and convergent validity. Once the multivariate normality was confirmed, we tested the factorial validity with maximum likelihood estimation. The model of the factor proposed is deemed valid when all items show a factorial load higher than 0.4 [44,47,48]. The construct’s validity was calculated through convergent validity (using the average variance extracted (AVE) for each factor and considering 0.50 as the minimum value) and the discriminant validity, confirmed by evidence that the AVE for each pair of factors is equal to or greater than the square of the correlation between them.

The statistical software IBM-SPSS Statistics version 26.0 and AMOS (IBM Corp, Armonk, NY, USA) were used to carry out all analyses.

### 2.4. Ethical Considerations

We collected data from the RN4CAST@pt study concerning primary health care after approval by the Research Ethics Committee of Universidade Católica Portuguesa—Porto (Ethics Clearance number 03/2018 and date of approval 14 May 2018). We assured the nurses who answered the RN4CAST Nurse Survey Instrument of the anonymity and privacy of data.

## 3. Results

Of a total of 1059 nurses, 85.8% were women. The average age was 43.5 years, with a standard deviation of 7.9 years. Graduates in nursing comprised 98.9% of the sample, and 1.1% had a bachelor’s degree. Specialist nurses comprised 54.7% of the sample. The average time working in the profession was 20.5 years, with a standard deviation of 7.8 years (Table 1).

### 3.1. Exploratory Factor Analysis

For exploratory factor analysis, Bartlett’s sphericity test was significant (*χ*^2^ = 378; *p* < 0.001), and the KMO index had a value of 0.901. Both are excellent values for the analysis of the main components, according to Almeida [45].

In the EFA, items 13, 14, and 22 did not meet the factor loading criteria of above 0.40, so they were excluded. The PES-NWI EFA in PHC identified five components that explain 53.5% of the total variance. The final solution consists of 28 items in five components: “Nurse Participation in Organization Affairs” (NPOA) with 11 items; “Nursing Foundations for Quality of Care” (NFQC) with six items; “Nurse Manager Ability, Leadership, and Support of Nurses” (NMALSN) with four items; “Staffing and Resource Adequacy” (SRA) with four items; and “Collegial Nurse–Physician Relations” (CNPR) with three items (Table 2). We deemed it necessary to maintain the perspective of Amaral et al. [19] and Lake [5]; thus, we kept the names of the components, only adjusting semantics or meaning in the NPOA dimension.

### 3.2. Reliability Analysis

The PES-NWI in primary health care showed very good reliability (α = 0.91) and a high level of internal scale consistency. The α score oscillated among dimensions (“Nurse Participation in Organization Affairs” with α = 0.87; “Nursing Foundations for Quality of Care” with α = 0.76; “Nurse Manager Ability, Leadership, and Support of Nurses” with α = 0.74; “Staffing and Resource Adequacy” with α = 0.75; “Collegial Nurse–Physician Relations” with α = 0.81 (Table 2)).

### 3.3. Confirmatory Factor Analysis

The CFA was carried out on the factorial structure of five PES-NWI factors found in our sample in PHC. Although the PES items showed good factor weights (>0.4), initially, the CFA model showed an inadequate fit (*χ*^2^/df = 2067.4; CFI = 0.845; GFI = 0.871; RMSEA = 0.069; MECVI = 2.08).

In addition, the Mahalanobis distances indicated the presence of several multivariate outliers, some of which were removed from the model, and the modification indexes were analyzed.

As the changes were not significant, this study analyzed the model modification indexes, with the largest occurring between the correlations of errors between items 17 and 5 and between items 27 and 23. When items belonging to the same factor present related errors, it is common to add this trajectory to the model, justifying it from the theoretical point of view by the similarity of formulation or content of the items.

**Figure 1:**
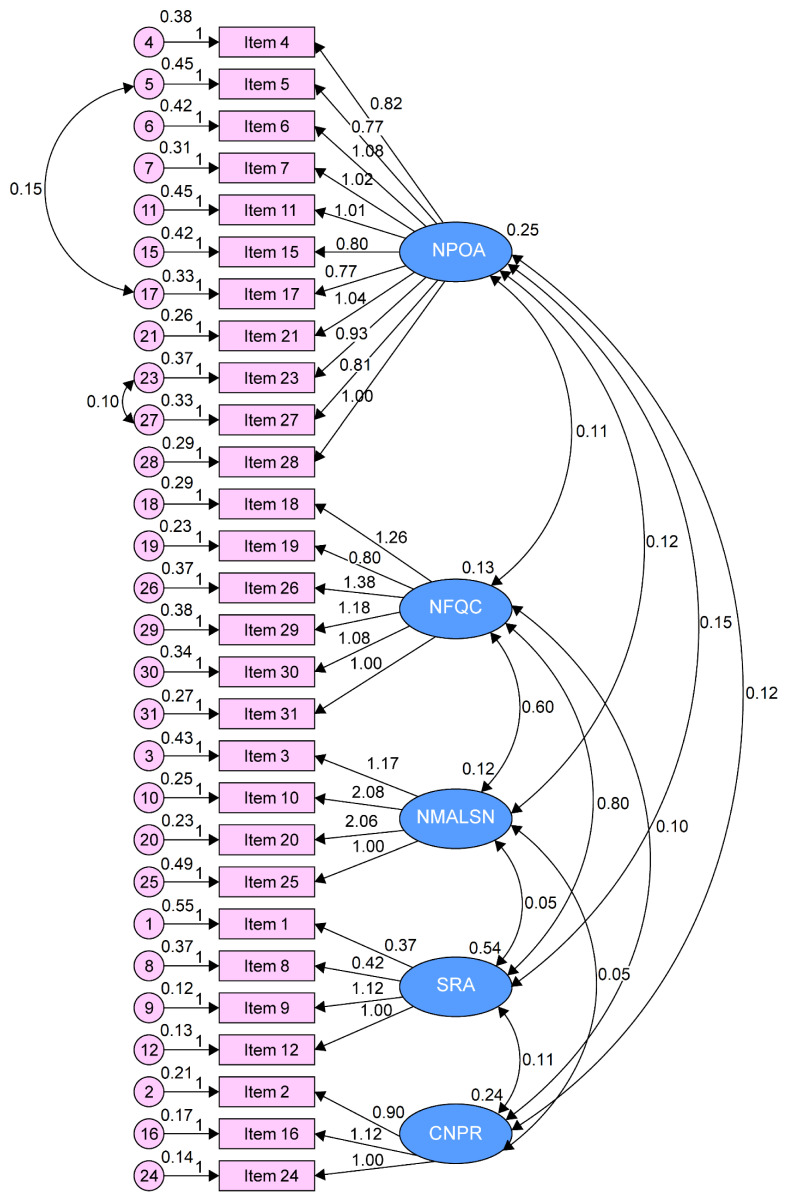
Five-factor model of the Practice Environment Scale of Nursing Work Index (PES-NWI) in primary health care in Portugal.

From the review, the model (Figure 1) showed a quality of fit with better indexes for the PHC sample and a lower MECVI (1.85 versus 2.08) than the initial model.

The construct reliability revealed an adequate internal consistency of the scale with a high level of internal consistency and good reproducibility of the full scale (α = 0.91) and subscales (Table 3). Considering VEM ≥ 0.50 [45,49] as an indicator of convergent validity, the scale proved to be adequate in all of its factors: “Nurse Participation in Organization Affairs”; “Nursing Foundations for Quality of Care”; “Nurse Manager Ability, Leadership, and Support of Nurses”; “Staffing and Resource Adequacy”; and “Collegial Nurse–Physician Relations”.

The analysis of the factorial invariance of the model in both independent subgroups (test and validation) showed adequate indexes of quality of fit in the final factorial solution (*χ*^2^/df = 1821.4; CFI = 0.867; GFI = 0.885; RMSEA = 0.064 (90% CI = 0.059–0.065, *p* = 0.003); SRMR = 0.058; MECVI = 1.85).

## 4. Discussion

This study aimed to examine the psychometric properties of the PES-NWI in primary health care in Portugal. This is the first validation study of the PES-NWI in primary health care in this country and the second internationally. These two reasons support the importance and relevance of this study at the national and international levels.

This study used data from RN4CAST@pt 2018 related to PHC in Portugal and is the first study with a large sample of nurses originating from all HCGs and health centers from the autonomous regions of Madeira and the Azores.

The results provide empirical evidence indicating the adequate psychometric performance of the PES-NWI in PHC in Portugal. The model, which has five components, is conceptually consistent with that suggested by the author of the original scale [5], based on exploratory and confirmatory analyses. However, the items of each component differ when compared with studies from other countries, which can be explained by differences in culture, health system models, organizational models, or nursing profession, which may influence nurses’ answers.

Studies in PHC have shown similarities in the composition of components, such as in the works of De Pedro-Gómez et al. [25,50], Gea-Caballero et al. [13], Parro-Moreno et al. [3,26], and Rabie et al. [28].

The final results of this study on the PES-NWI in PHC correspond exactly to two dimensions of Lake’s original version [5] and the Portuguese version of Amaral et al. [19]: “Staffing and Resource Adequacy” and “Collegial Nurse–Physician Relations”.

The items excluded (13, 14, and 22) by the factor analysis belong to the dimensions “Nursing Foundations for Quality of Care” and “Nurse Manager Ability, Leadership, and Support of Nurses” in Lake’s original version [5] and in the Portuguese version of Amaral et al. [19]. Thus, this study used a slightly shorter version of the PES-NWI that Lake proposed [16].

The CFA showed that the five-factor model is the most stable and well represents this study’s large sample of data in PHC in Portugal, as it is similar to the one presented by Lake, the author of the original scale [5].

All coefficients were significant. The coefficients of the model fit indexes were satisfactory and supported the five-factor factorial structure for PHC. 

In addition, analysis of the five-factor model’s invariance confirmed its stability. The high value of internal consistency observed in the five-factor model of our study (α = 0.91) is greater than the original values obtained by Lake [5] (α = 0.82) and by Amaral et al. [19] (α = 0.89) and equal to that found by De Pedro-Gómez et al. [25] (α = 0.91).

Furthermore, there is evidence of acceptable reliability and validity with adequate internal consistency in the full scale and subscales of the model. The results agree with other studies on the psychometric properties of the PES-NWI, but in different organizational contexts in health [5,18,19,21,31,32,35,36,39,40,41,42,50,51,52,53,54,55].

### Limitations

This study was carried out based on the current organizational context of PHC in the country resulting from the 2005 reformation, in which health centers merged into significant organizational structures. Therefore, studies should be performed in each HCG and compared with our results to evaluate the NPE in each PHC organization.

The relationship and the impact between the NPE and the quality of care, patient safety care, workload and endowments, job satisfaction and intention to leave, and emotional exhaustion were not studied. Therefore, further studies must be performed in these areas. One important topic of future study is communication and information technologies in the PES-NWI.

## 5. Conclusions

This study, which was carried out with a large sample of Portuguese nurses from PHC within the scope of the RN4CAST@pt 2018 study, provided psychometric evidence and revealed an adequate five-factor structure of the PES-NWI. The evidence supports the reliability and validity of the structure and agrees with the structure suggested by the author of the original scale. All factors showed appropriate factor loadings and had a semantic relationship.

This study shows that, in the Portuguese nursing context of PHC, the PES-NWI is a relevant instrument that can support decision-making by nurse managers, can characterize the NPE, and can improve work conditions in PHC organizations. The PES-NWI in PHC enables the classification of environments as favorable or unfavorable to patients’ safety and health and to the quality of the nursing care provided. The results showed that the scale is valid and can be used in clinical practice, nursing management, and PHC investigations. This study also demonstrated the international relevance of the use of the PES-NWI in PHC, as only a single study is available at present.

This study offers valuable contributions to the strategic planning and health policies in PHC contexts.

## Figures and Tables

**Table 1:** Descriptive analysis of the demographic characteristics data.

	*N*	%	Mean	SD
Gender	-	-	-	-
Female	909	85.8	-	-
Male	150	14.2	-	-
Academic Degree	-	-	-	-
Graduates	1.047	98.9	-	-
Bachelor’s	12	1.1	-	-
Specialists	-	-	-	-
Specialist nurses	579	54.7	-	-
Age	-	-	43.5	7.9
Years of nursing	-	-	20.5	7.8

**Table 2:** The Practice Environment Scale of Nursing Work Index (PES-NWI) components in primary health care.

ITEMS	COMPONENTS				
	Nurse Participation in Organization Affairs	Nursing Foundations for Quality of Care	Nurse Manager Ability, Leadership, and Support of Nurses	Staffing and Resource Adequacy	Collegial Nurse–Physician Relations
Item 4	0.51	-	-	-	-
Item 5	0.67	-	-	-	-
Item 6	0.67	-	-	-	-
Item 7	0.57	-	-	-	-
Item 11	0.44	-	-	-	-
Item 15	0.53	-	-	-	-
Item 17	0.67	-	-	-	-
Item 21	0.58	-	-	-	-
Item 23	0.64	-	-	-	-
Item 27	0.59	-	-	-	-
Item 28	0.52	-	-	-	-
Item 18	-	0.45	-	-	-
Item 19	-	0.52	-	-	-
Item 26	-	0.59	-	-	-
Item 29	-	0.69	-	-	-
Item 30	-	0.67	-	-	-
Item 31	-	0.76	-	-	-
Item 3	-	-	0.53	-	-
Item 10	-	-	0.83	-	-
Item 20	-	-	0.79	-	-
Item 25	-	-	0.41	-	-
Item 1	-	-	-	0.49	-
Item 8	-	-	-	0.59	-
Item 9	-	-	-	0.87	-
Item 12	-	-	-	0.87	-
Item 2	-	-	-	-	0.80
Item 16	-	-	-	-	0.81
Item 24	-	-	-	-	0.76
Explained Variance	28.9	6.7	8	5.2	4.7
Cronbach’s alpha	0.87	0.76	0.74	0.75	0.81

**Table 3:** Analysis of the construct validity of the PES-NWI in primary health care. AVE, average variance extracted.

Components	Alpha	AVE
1. Nurse Participation in Organization Affairs	0.87	0.88
2. Nursing Foundations for Quality of Care	0.76	1.28
3. Nurse Manager Ability, Leadership, and Support of Nurses	0.74	2.75
4. Staffing and Resource Adequacy	0.75	0.59
5. Collegial Nurse–Physician Relations	0.81	1.02

## Data Availability

Restrictions apply to the availability of these data. Data was obtained from third party and are available with the permission of third party.
